# Editorial: Hip arthroscopy: Pathologies, surgical techniques and complications

**DOI:** 10.3389/fsurg.2022.1110716

**Published:** 2023-01-11

**Authors:** Carlos Suarez-Ahedo

**Affiliations:** Adult Hip & Knee Reconstruction Department, National Institute of Rehabilitation Luis Guillermo Ibarra Ibarra, Tlalpan, Mexico

**Keywords:** hip, arthroscopy, arthroplasty, hip joint, hip pain

**Editorial on the Research Topic** Hip arthroscopy: Pathologies, surgical techniques and complications

In the beginning, hip arthroscopy was mainly diagnostic, but a better understanding of the pathology, better examination techniques, and better imaging have led to increasing numbers of therapeutic procedures being performed and also led to the recognition of new pathologies, in a case report **Jian et al.** will show a patient with lateral hip pain who failed with conservative treatment, hip endoscopy was performed on this patient, resulting in significant improvement to regular daily and social activities in the mid-term.

Hip arthroscopy has been recognized as a surgical technique to treat bone deformity, periarticular soft tissue pathologies, and complications. However, it is important to recognize that OA and ONFH can be induced by exosomes which may play an important role as an inducer and serve as a promising treatment for early intervention, just like **LV et al.** showed us in its interesting review. The original research by **Yu et al.** demonstrates that there is a strong association between blood lipid metabolism and coagulation function with IONFH, which opens the door for future research.

Unfortunately, when the damage around the Hip is severe, a total hip replacement can be the only available treatment option that we can offer. as we already know, total hip replacement is the most successful and cost-effective orthopedic surgery, however, leg length discrepancy is one of the most common causes of a lawsuit after THR in the U.S.A. that is why the study presented by **Chen et al.** is important because they show that the horizontal calibrator provides more accurate limb length and femoral offset. however, when THR fails, one of its causes can be secondary to an acetabular defect, and a revision THR will be needed. The Systematic Review by **Cheng et al.** showed that using a jumbo cup is a recommended method for acetabular reconstruction in rTHA with satisfying clinical outcomes and survivorship.

Regarding surgical techniques, Li et al. presented an RCS that shows the importance of reconstructing the joint capsule and conjoint tendon to enhance muscle strength for the patient's best recovery. In its case report, **Gebhardt et al.** show us the importance of capsule closure to avoid iliopsoas tendon entrapment intraarticular.

The remaining article by **Shen et al.** looks into postoperative delirium in the geriatric population after hip surgery. The study proposes a prediction score that will enable delirium risk stratification for hip fracture patients and facilitate the development of strategies for delirium prevention.

This article series provides the most current information in a rapidly evolving field, according to the editorial board of Hip Arthroscopy: Pathologies, surgical techniques, and complications. In this series of articles, we will discuss hip pathology and the various treatment options available.

